# Etiology of hormone receptor positive breast cancer differs by levels of histologic grade and proliferation

**DOI:** 10.1002/ijc.31352

**Published:** 2018-03-25

**Authors:** Mustapha Abubakar, Jenny Chang‐Claude, H. Raza Ali, Nilanjan Chatterjee, Penny Coulson, Frances Daley, Fiona Blows, Javier Benitez, Roger L. Milne, Hermann Brenner, Christa Stegmaier, Arto Mannermaa, Anja Rudolph, Peter Sinn, Fergus J. Couch, Peter Devilee, Rob A.E.M. Tollenaar, Caroline Seynaeve, Jonine Figueroa, Jolanta Lissowska, Stephen Hewitt, Maartje J. Hooning, Antoinette Hollestelle, Renée Foekens, Linetta B. Koppert, kConFab Investigators, Manjeet K. Bolla, Qin Wang, Michael E. Jones, Minouk J. Schoemaker, Renske Keeman, Douglas F. Easton, Anthony J. Swerdlow, Mark E. Sherman, Marjanka K. Schmidt, Paul D. Pharoah, Montserrat Garcia‐Closas

**Affiliations:** ^1^ Division of Cancer Epidemiology and Genetics National Cancer Institute, National Institutes of Health Rockville MD; ^2^ Division of Genetics and Epidemiology The Institute of Cancer Research London United Kingdom; ^3^ Division of Cancer Epidemiology German Cancer Research Center (DKFZ) Heidelberg Germany; ^4^ University Cancer Center Hamburg, University Medical Center Hamburg‐Eppendorf Hamburg Germany; ^5^ Cancer Research UK Cambridge Institute, University of Cambridge Cambridge United Kingdom; ^6^ Department of Biostatistics Bloomberg School of Public Health, Johns Hopkins University Baltimore MD; ^7^ Department of Oncology, School of Medicine, Sidney Kimmel Comprehensive Cancer Center Johns Hopkins University Baltimore MD; ^8^ Division of Breast Cancer Research, Breast Cancer Now Toby Robins Research Centre The Institute of Cancer Research London United Kingdom; ^9^ Department of Oncology, Centre for Cancer Genetic Epidemiology University of Cambridge Cambridge United Kingdom; ^10^ Human Genetics Group, Human Cancer Genetics Program, Spanish National Cancer Research Centre (CNIO) Madrid Spain; ^11^ Centro de Investigacion en Red de Enfermedades Raras (CIBERER) Valencia Spain; ^12^ Cancer Epidemiology Centre, Cancer Council Victoria Melbourne VIC Australia; ^13^ Melbourne School of Population and Global Health, Centre for Epidemiology and Biostatistics The University of Melbourne Melbourne VIC Australia; ^14^ Division of Clinical Epidemiology and Aging Research German Cancer Research Center (DKFZ) Heidelberg Germany; ^15^ Division of Preventive Oncology German Cancer Research Center (DKFZ), and National Center for Tumor Diseases (NCT) Heidelberg Germany; ^16^ German Cancer Consortium (DKTK), German Cancer Research Center (DKFZ) Heidelberg Germany; ^17^ Saarland Cancer Registry Saarland Germany; ^18^ School of Medicine Institute of Clinical Medicine, Pathology and Forensic Medicine, Cancer Center of Eastern Finland, University of Eastern Finland Kuopio Finland; ^19^ Department of Clinical Pathology, Imaging Center Kuopio University Hospital Kuopio Finland; ^20^ Department of Pathology Institute of Pathology, Heidelberg University Hospital Heidelberg Germany; ^21^ Department of Laboratory Medicine and Pathology Mayo Clinic Rochester MN; ^22^ Department of Human Genetics & Department of Pathology Leiden University Medical Center Leiden The Netherlands; ^23^ Department of Surgery Leiden University Medical Center Leiden The Netherlands; ^24^ Department of Medical Oncology Family Cancer Clinic, Erasmus MC Cancer Institute Rotterdam The Netherlands; ^25^ Usher Institute of Population Health Sciences and Informatics, The University of Edinburgh Scotland United Kingdom; ^26^ Department of Cancer Epidemiology and Prevention M. Sklodowska‐Curie Memorial Cancer Center and Institute of Oncology Warsaw Poland; ^27^ Laboratory of Pathology National Cancer Institute, National Institutes of Health Rockville MD; ^28^ Department of Surgical Oncology Erasmus MC Cancer Institute Rotterdam The Netherlands; ^29^ Research Department Peter MacCallum Cancer Centre Melbourne VIC Australia; ^30^ The Sir Peter MacCallum Department of Oncology University of Melbourne, Parkville Melbourne VIC Australia; ^31^ Department of Public Health and Primary Care, Centre for Cancer Genetic Epidemiology University of Cambridge Cambridge United Kingdom; ^32^ Division of Molecular Pathology Netherlands Cancer Institute, Antoni van Leeuwenhoek Hospital Amsterdam The Netherlands; ^33^ Division of Breast Cancer Research The Institute of Cancer Research London United Kingdom; ^34^ Division of Epidemiology, Department of Health Sciences Research Mayo Clinic Jacksonville FL; ^35^ Division of Psychosocial Research and Epidemiology Netherlands Cancer Institute, Antoni van Leeuwenhoek Hospital Amsterdam The Netherlands

**Keywords:** breast cancer, epidemiology, obesity, nulliparity, hormone therapy, grade, KI67, proliferation

## Abstract

Limited epidemiological evidence suggests that the etiology of hormone receptor positive (HR+) breast cancer may differ by levels of histologic grade and proliferation. We pooled risk factor and pathology data on 5,905 HR+ breast cancer cases and 26,281 controls from 11 epidemiological studies. Proliferation was determined by centralized automated measures of KI67 in tissue microarrays. Odds ratios (OR), 95% confidence intervals (CI) and *p*‐values for case–case and case–control comparisons for risk factors in relation to levels of grade and quartiles (Q1–Q4) of KI67 were estimated using polytomous logistic regression models. Case–case comparisons showed associations between nulliparity and high KI67 [OR (95% CI) for Q4 *vs*. Q1 = 1.54 (1.22, 1.95)]; obesity and high grade [grade 3 *vs*. 1 = 1.68 (1.31, 2.16)] and current use of combined hormone therapy (HT) and low grade [grade 3 *vs*. 1 = 0.27 (0.16, 0.44)] tumors. In case–control comparisons, nulliparity was associated with elevated risk of tumors with high but not low levels of proliferation [1.43 (1.14, 1.81) for KI67 Q4 *vs*. 0.83 (0.60, 1.14) for KI67 Q1]; obesity among women ≥50 years with high but not low grade tumors [1.55 (1.17, 2.06) for grade 3 *vs*. 0.88 (0.66, 1.16) for grade 1] and HT with low but not high grade tumors [3.07 (2.22, 4.23) for grade 1 *vs*. 0.85 (0.55, 1.30) for grade 3]. Menarcheal age and family history were similarly associated with HR+ tumors of different grade or KI67 levels. These findings provide insights into the etiologic heterogeneity of HR+ tumors.

## Abstract 2

**What's new?**

Limited epidemiological evidence suggests that the etiology of hormone receptor positive (HR+) breast cancer may differ by HR+ tumor subtypes as defined by histologic grade and proliferation level. In this report pooling risk factor data from a consortium of breast cancer studies, the authors found associations between nulliparity and highly proliferative tumors; obesity and high grade tumors; and current use of combined hormone therapy and low grade tumors. These results provide insights into heterogeneity of HR+ tumors that may be reflective of differences in etiological pathways, and could also have implications for risk prediction of aggressive subtypes of HR+ tumors.

**Abbreviations**
BCAC: Breast Cancer Association Consortium
BMI: body mass index
ER: estrogen receptor
HR+: hormone receptor positive
HT: hormone therapy
ICR: Institute of Cancer Research, London
NGS: Nottingham grading system
OR: odds ratio
PR: progesterone receptor
TMA: tissue microarray

## Introduction

Breast cancer is a heterogeneous disease at the morphological, molecular and genomic level, defining subtypes with distinct biological and clinical behavior.^1^, ^2^, ^3^ Expression of hormone receptors (HR; i.e., estrogen receptor (ER) or progesterone receptor (PR)) distinguishes two classes of tumors thought to derive from different cells of origin: HR+ tumors deriving from luminal epithelial cells and HR− from basal/myoepithelial cells.^1^ In Western populations, HR+ tumors occur more commonly (∼70% of tumors) and have a later age at onset and better short‐term prognosis than HR− tumors.^4^, ^5^ While epidemiological studies have shown that these two subtypes may have distinct risk factor associations,^6^, ^7^, ^8^, ^9^ little is known about etiologic heterogeneity within HR+ tumors.^10^, ^11^

Histologic grade is an important indicator of tumor aggressiveness that reflects three features including tubule formation, nuclear pleomorphism and mitotic count, which is directly related to proliferation.^12^ Due to the latter feature, it is highly correlated with KI67 (a marker of proliferation) and both have been used to identify surrogates for two HR+ tumors identified by expression tumor profiling studies, i.e. luminal A and luminal B subtypes.^13^, ^14^, ^15^ Epidemiological studies suggest that these two subtypes could have differential associations with risk factors.^10^, ^16^ However, although correlated, histologic grade and KI67 reflect different biological features of tumors that could be of etiological relevance. Unlike grade which encompasses both differentiation and proliferation, KI67 is expressed only during the proliferative phases of the cell‐cycle and is one of the most commonly used markers of proliferation.^17^, ^18^, ^19^ Its function is not fully understood but it is thought to mediate assembly of the peri‐chromosomal compartment in human cells.^20^

Accumulating epidemiological data suggest that breast cancer risk factors may be distinctly associated with grade and KI67.^21^, ^22^, ^23^ Three previous studies found associations between high BMI and high levels of histologic grade but not KI67^21^, ^22^, ^23^ whilst younger age at onset of breast cancer and being of African‐American ethnicity were reportedly associated with high levels of KI67 but not histologic grade.^23^ These studies were case‐series with limited sample sizes (346–668 cases), and were based on semi‐quantitative visual scores for KI67. This scoring approach is characterized by poor inter‐observer reproducibility^24^, ^25^ and offers limited opportunities for evaluating dose–response relationships. Thus, studies with larger sample sizes and standardized quantitative measures of KI67 across studies are needed to evaluate the relationship between breast cancer risk factors and HR+ tumors defined by their levels of proliferation and histologic grade.

In this report, we pooled risk factor data from a consortium of breast cancer studies to examine the relationship of breast cancer risk factors with subtypes of HR+ tumors defined by levels of histologic grade and KI67 expression, determined by centralized automated scoring of tissue microarrays (TMAs) as previously described.^26^

## Materials and Methods

### Study population

A total of 5,905 HR+ invasive breast cancer cases and 26,281 controls were pooled from 11 epidemiological case–control studies with TMAs and risk factor information in the Breast Cancer Association Consortium (BCAC). Study populations were from Europe, Australia and North America. Details of the contributing studies including designs, country of location, method of recruitment, age range, sources and eligibility of cases and controls are provided in Supporting Information Table S1. In brief, this analysis comprised 11 case–control studies (one of them (UKBGS) nested within a prospective cohort study). Six studies (CNIO, MCBCS, ORIGO, RBCS, SEARCH and kConFab) were of hospital‐based or mixed study designs (considered “non‐population‐based” studies), whilst five studies (ESTHER, KBCP, MARIE, PBCS and UKBGS) were population‐based. All participants in each of the study groups provided written informed consent and all studies gained approval from local ethics committees.

### Risk factors

Data on risk factors were derived from questionnaires that were administered to participants at recruitment in each of the participating BCAC studies. Harmonization, central querying and quality checks on these data were performed by investigators at the German Cancer Research Institute, Heidelberg. The current analysis included risk factors for which there is evidence in the literature to suggest a heterogeneous relationship with clinicopathological characteristics and for which we had data. In this regard, five risk factors were identified – age at menarche, parity, body mass index (BMI), use of combined hormone therapy (HT) and family history of breast cancer. Supporting Information Table S2 shows the number of cases and controls from each study with risk factor information.

### Pathological characteristics

Data on hormone receptor status were obtained from clinical records. Levels of histologic grade were assigned by local study pathologists in the respective study groups. Tumors were graded as 1 (low grade or well‐differentiated), 2 (intermediate grade or moderately differentiated) and 3 (high grade or poorly differentiated). The extent of proliferation in breast cancer tissues was determined using measures of KI67. Scores were centrally generated at the Institute of Cancer Research (ICR) in London by using a digital image analysis protocol that was developed for the quantification of KI67 in breast cancer TMAs as previously described.^26^ In brief, a total of 166 TMAs were collected for evaluation from the participating BCAC studies. These were stained using a standard protocol of (Dako, Cheshire UK) MIB‐1 antibody diluted 1/50 and visualized using the Dako REAL kit (K5001). Automated scoring was performed using the Ariol machine (Leica Biosystems, Newcastle UK), which has functionality that allows for the discrimination of malignant and non‐malignant nuclei using shape and size characteristics as well as the automatic detection of KI67 positive and negative malignant nuclei using color deconvolution. The algorithm was used to generate quantitative (0–100% positive cells) KI67 scores. As previously reported,^26^ Ariol scores showed good agreement with standardized pathologist's scores. Subsequently, automated KI67 scores were merged with other risk factor and pathological characteristics. The majority of the 5,905 cases had complete data on KI67 (83%) or grade (76%) and at least one risk factor (see Supporting Information Table S3 for details). All pathology data were harmonized and quality checked by investigators at the Netherlands Cancer Institute, Amsterdam.

### Statistical analysis

Participant ages at diagnosis/ages at interview were categorized into five classes (<40, 40–49, 50–59, 60–69 and ≥70). Age at menarche was categorized into four classes (≤12, 13, 14, ≥15). Parity was defined as nulliparous or parous for case–case and case–control comparisons. For BMI, three well‐defined categories were used (normal <25 kg/m^2^; overweight 25–30 kg/m^2^ and obese >30 kg/m^2^) and the case–control analysis was conducted for groups of women stratified according to age (<50 years and ≥50 years) as a surrogate for menopausal status. This was done to account for previously reported differences in the association between BMI and breast cancer risk by menopausal status. For the case–case comparisons, BMI was not differentially related to tumor grade/KI67 levels by age categories (proxy for menopausal status); as a result, case–case analysis was not stratified according to age. HT use was categorized into those who never used HT, former users and current users. Due to very small numbers of those who reported using estrogen only formulations, our analysis involved only those women who took combined estrogen and progesterone formulations. Family history of breast cancer in a first‐degree relative was categorized as yes (if present) or no (if absent). Frequency tables were used to assess the distribution of the risk factors among cases and controls stratified by study design. To test for differences in the distribution of risk factors for cases and controls by study design, we created a dummy variable for design and modeled this as the outcome with the different risk factors as predictors. Box plots and nonparametric Kruskal–Wallis equality of median test were used to assess the distribution of KI67 across categories of histologic grade, overall and by study.

We constructed a polytomous unconditional logistic regression model for each risk factor variable, and performed case–case and case–control comparisons within the same model. For case–case comparisons, odds ratios (OR), 95% confidence intervals and *p*‐values for the associations between breast cancer risk factors [menarche (≤12 *vs*. ≥15 years); parity (nulliparous *vs*. parous); BMI (25–30 kg/m^2^ and >30 kg/m^2^ *vs*. <25 kg/m^2^, respectively); HT (former and current *vs*. never, respectively); family history (yes *vs*. no)] and quartiles of KI67 [Q1 (base category), <25th percentile (0–1.49%); Q2, 25–50th percentile (1.50–4.29%); Q3, >50–75th percentile (4.30–10.40%); Q4, >75th percentile (>10.40%)] and histologic grade [grades 1 (base category), 2, 3] were estimated. For case–control comparisons, an interaction term between study design (population‐based *vs*. non‐population‐based) and the risk factor of interest was included to obtain estimates of association by study design. Because of previously reported biases in case–control ORs estimated from non‐population‐based studies,^9^ only case–control ORs from population‐based studies are presented in tables. However, ORs for case–case comparisons and corresponding tests are based on data from all cases (i.e., from both population‐based and non‐population‐based studies). Meta‐analyses of study‐specific case–case and case–control ORs were performed to test for between‐study heterogeneity in the OR estimates.

We examined dose–response relationships between risk factors and levels of KI67, by using the median % positive cells in each quartile of KI67 as constraints in an ordered polytomous logistic regression model.^27^ To determine if the relationships between nulliparity, obesity and current use of combined HT are distinct with respect to grade and KI67, we applied a 2‐stage meta‐regression model.^28^ In the first stage of the 2‐stage meta‐regression model, we performed a polytomous logistic regression analyses for subtypes of HR+ breast cancer defined by cross‐classification of levels (Q1–Q4) of KI67 and histologic grade (low (grade1) and high (grades 2 and 3)). In the second stage, we modeled the subtype‐specific log odds ratios and standard errors using KI67 and grade. This approach allowed us to evaluate if the risk factor‐subtype associations are different across subtypes defined by KI67 whilst controlling for grade, and vice versa. Also, by including an interaction term between KI67 and grade we were able to examine if the relationship between risk factors and subtypes defined by levels of KI67 were modified by grade or vice versa.

Analysis on each risk factor was limited to studies that provided information on that risk factor. Missing values were addressed by creating indicators for missing values in our models. As sensitivity analysis, all risk factors were mutually adjusted for in a multivariate model comprising data from three studies with information on the five risk factors that were evaluated. All analyses, including case–case and case–control comparisons, were adjusted for age and study. All statistical tests were two‐sided and performed using Stata statistical software version 13.1.

## Results

Table 1 shows a description of the characteristics of the study participants based on population‐based (*N* = 5 studies) and non‐population‐based (*N* = 6 studies) designs. While the distribution of most risk factors in cases was similar by study design, most risk factors showed different distributions in population and non‐population‐based studies.

**Table 1:** Characteristics of cases and controls in population and non‐population based studies

	Population‐based				Non‐population‐based			
Characteristic	Controls (no.)	%	Cases (no.)	%	Controls (no.)	%	Cases (no.)	%
Age, years								
<40	252	2.2	42	2.1	590	4.7	249	6.7
40–49	1,247	10.9	293	14.4	2,135	17.0	905	24.3
50–59	3,999	34.9	682	33.6	4,696	37.4	1,456	39.1
60–69	4,720	41.1	708	34.9	3,662	29.2	882	23.7
≥70	1,256	10.9	303	14.9	1,464	11.7	233	6.3
Age at menarche, years								
≤12	2,140	26.2	522	27.8	3,491	40.5	1,123	42.9
13	1,838	22.5	431	22.9	2,263	26.3	640	24.4
14	2,034	24.9	498	26.5	1,617	18.8	471	18.0
≥15	2,168	26.5	427	22.7	1,245	14.4	385	14.7
Parity								
None	1,221	13.5	310	15.6	1,437	16.4	396	14.3
1–2	5,941	65.6	1,331	66.9	4,495	51.3	1,484	53.5
3–4	1,726	19.0	312	15.7	2,460	28.1	798	28.8
≥5	175	1.9	37	1.9	363	4.1	94	3.4
BMI, kg/m^2^								
*Among women <50 years*								
<25	542	44.4	194	51.1	672	49.7	461	52.3
25–30	431	35.3	144	37.9	387	28.6	273	31.0
>30	249	20.4	42	11.1	292	21.6	147	16.7
*Among women ≥ 50 years*								
<25	2,775	35.6	472	29.5	1,998	34.5	646	37.4
25–30	3,028	38.9	641	40.1	2,366	40.9	679	39.3
>30	2,005	25.7	486	30.4	1,419	24.5	401	23.2
Combined HT Use								
Never	4,836	70.7	1,000	73.4	1,070	74.9	196	75.1
Former	849	12.4	117	8.6	238	16.7	29	11.1
Current	1,154	16.9	245	18.0	120	8.4	36	13.8
Family history								
No	8,023	90.2	1,707	87.4	7,778	88.6	1,997	76.9
Yes	874	9.8	247	12.6	1,004	11.4	599	23.1

The study population comprised 11 studies participating in the Breast Cancer Association Consortium (see Supporting Information Table S1 for details of the individual studies) with population (ESTHER, KBCP, MARIE, PBCS, UKBGS) and non‐population (CNIO, kConFab, MCBCS, ORIGO, RBCS, SEARCH) based designs. In a model with study design as the outcome: for controls, the distribution of all the risk factors differed by design (*p*‐value <0.05); for cases, only menarche and family history were different by design.

Overall, the median and mean positive cells stained for KI67 was 4.2% and 8.2%, respectively. Most tumors were of intermediate grade (52%), followed by low grade (26%) and high grade (22%) tumors. As expected, grade 1 tumors had lower KI67 scores compared to grades 2 and 3 tumors [median and mean = 3% and 6.3%; 4.3% and 8%; 7% and 11% for grades 1, 2 and 3 tumors, respectively]. A similar pattern of association between KI67 and histologic grade was seen across studies (Supporting Information Fig. S1).

### Case–case comparisons for the associations between breast cancer risk factors and HR+ tumors defined by levels of histologic grade and KI67

As shown in Table 2, we observed that compared to their normal weight counterparts, tumors occurring amongst overweight and obese women were more likely to be of higher (grades 2 and 3) than lower (grade 1) grade. Specifically, we observed overweight women to have 33% (95% CI = 1.13, 1.58) and 23% (95% CI = 1.00, 1.52) increased odds of developing grades 2 and 3 than grade 1 tumors, respectively. Similarly, high grade tumors were more likely to occur amongst obese than normal weight women [*vs*. grade 1, OR (95% CI) = 1.67 (1.13, 2.05); *p*‐value = 0.001 for grade 2 and 1.68 (1.31, 2.16); *p*‐value = <0.001 for grade 3 tumors]. As shown in Supporting Information Table S4, these associations were similar following stratification by tumor size (*p*‐value for interaction (*p*__interaction_) = 0.52).

**Table 2:** Case–case odds ratios and 95% CI for the associations between breast cancer risk factors and subtypes of HR+ tumors defined by levels of histologic grade

	Histologic grade*						
	Grade 1 (comparison group)	Grade 2			Grade 3		
Risk factor	*N*	*N*	OR (95% CI)	*p*‐Value	*N*	OR (95% CI)	*p*‐Value
Menarche							
≥15 years	183	417	1.00 (referent)		157	1.00 (referent)	
14 years	218	497	1.01 (0.80, 1.28)	0.93	186	0.99 (0.74, 1.33)	0.97
13 years	266	529	0.96 (0.76, 1.20)	0.71	192	0.89 (0.67, 1.18)	0.42
≤12 years	363	836	1.09 (0.87, 1.35)	0.46	299	0.96 (0.73, 1.26)	0.77
Parity							
Parous	902	2,089	1.00 (referent)		745	1.00 (referent)	
Nulliparous	165	322	0.86 (0.70, 1.06)	0.16	157	1.09 (0.86, 1.40)	0.46
BMI							
<25 kg/m^2^	454	832	1.00 (referent)		332	1.00 (referent)	
25–30 kg/m^2^	385	929	**1.33 (1.13, 1.58)**	**0.001**	326	**1.23 (1.00, 1.52)**	**0.05**
>30 kg/m^2^	202	596	**1.67 (1.13, 2.05)**	**<0.0001**	212	**1.68 (1.31, 2.16)**	**<0.0001**
Combined HT use							
Never	169	545	1.00 (referent)		156	1.00 (referent)	
Former	33	76	0.71 (0.45, 1.12)	0.15	17	0.47 (0.25, 0.89)	**0.02**
Current	84	134	**0.45 (0.32, 0.63)**	**<0.0001**	29	**0.27 (0.16, 0.44)**	**<0.0001**
Family history							
No	844	1,918	1.00 (referent)		399	1.00 (referent)	
Yes	183	399	1.03 (0.83, 1.28)	0.78	173	1.07 (0.82, 1.40)	0.61

*Histologic grade (1 = low/well‐differentiated; 2 = intermediate/moderately differentiated; 3 = high/poorly differentiated). ORs and corresponding tests are based on data from all cases i.e. both population and non‐population‐based. All models were adjusted for age and study and no evidence was observed of between‐study heterogeneity in study‐specific OR estimates for BMI (*p*‐value = 0.96) and HRT (*p*‐value = 0.95).

Statistically significant *p*‐values are indicated in bold.

Compared to women who never took HT, tumors occurring amongst current users of combined HT were less likely to be high than low grade [*vs*. grade 1: OR (95% CI) = 0.45 (0.32, 0.63); *p*‐value = <0.001 for grade 2 and 0.27 (0.16, 0.44); *p*‐value = <0.001 for grade 3 tumors]. When we tested the associations between tumor grade, KI67 and morphology (ductal *vs*. lobular) in relation to HT use, all three tumor features were associated with HT use in univariate models at *p*‐value <0.05. However, following mutual adjustment for all three features in a multivariable model, only histologic grade remained associated with HT use (OR (95% CI) = 0.45 (0.27, 0.76); *p*‐value = 0.003 for grades 2 *vs*. 1 and 0.25 (0.11, 0.57); *p*‐value = 0.001 for grades 3 *vs*. 1). Furthermore, as shown in Supporting Information Table S4, HT use remained associated with low grade tumors regardless of tumor size (*p*__interaction_ = 0.78). Age at menarche, nulliparity and family history of breast cancer in a first‐degree relative were not differentially related to HR+ tumors defined by levels of histologic grade.

As shown in Table 3, compared to tumors occurring among parous women, those occurring among nulliparous women were more likely to have higher KI67 expression and a statistically significant gradient was observed in this relationship [OR (95% CI) *vs*. KI67 Q1 = 1.14 (1.06, 1.23) for KI67 Q2; 1.22 (1.09, 1.37) for KI67 Q3 and 1.50 (1.20, 1.88) for KI67 Q4; *p*‐value for trend 0.001]. There was weaker or no evidence for associations with KI67 levels for age at menarche, BMI, HT and family history of breast cancer in a first‐degree relative.

**Table 3:** Case–case odds ratios and 95% CI for the associations between breast cancer risk factors and subtypes of HR+ tumors defined by levels of tumor proliferation indicated by KI67

	KI67*										
	Q1 (comparison group)	Q2			Q3			Q4			
Risk factor	*N*	*N*	OR (95% CI)	P‐Value	*N*	OR (95% CI)	*p*‐Value	*N*	OR (95% CI)	*p*‐Value	*p*_trend
Menarche											
≥15 years	206	209	1.00 (referent)		196	1.00 (referent)		201	1.00 (referent)		
14 years	236	263	1.11 (0.85, 1.14)	0.45	244	1.07 (0.82, 1.41)	0.60	226	0.96 (0.73, 1.26)	0.76	0.49
13 years	302	253	0.85 (0.65, 1.10)	0.22	262	0.96 (0.74, 1.25)	0.75	254	0.94 (0.72, 1.22)	0.65	0.99
≤12 years	450	401	0.96 (0.75, 1.22)	0.75	384	1.01 (0.78, 1.29)	0.96	410	1.10 (0.86, 1.41)	0.44	0.30
Parity											
Parous	1,119	1,011	1.00 (referent)		976	1.00 (referent)		950	1.00 (referent)		
Nulliparous	158	183	**1.29 (1.03, 1.64)**	**0.03**	175	**1.30 (1.03, 1.65)**	**0.03**	190	**1.54 (1.22, 1.95)**	**<0.0001**	**0.001**
BMI											
<25 kg/m^2^	483	477	1.00 (referent)		411	1.00 (referent)		412	1.00 (referent)		
25–30 kg/m^2^	504	418	0.79 (0.65, 0.95)	**0.01**	408	0.87 (0.72, 1.05)	0.16	419	0.86 (0.72, 1.05)	0.14	0.53
>30 kg/m^2^	256	250	0.86 (0.69, 1.07)	0.18	288	1.05 (0.85, 1.31)	0.64	285	0.99 (0.79, 1.24)	0.93	0.67
Combined HT use											
Never	151	192	1.00 (referent)		269	1.00 (referent)		273	1.00 (referent)		
Former	37	34	0.96 (0.57, 1.62)	0.88	35	0.81 (0.48, 1.36)	0.43	23	0.65 (0.37, 1.16)	0.14	0.12
Current	72	78	1.11 (0.74, 1.66)	0.60	56	0.66 (0.43, 1.00)	**0.05**	51	0.68 (0.44, 1.05)	0.09	**0.03**
Family history											
No	907	923	1.00 (referent)		940	1.00 (referent)		959	1.00 (referent)		
Yes	266	226	0.95 (0.76, 1.21)	0.71	182	0.89 (0.70, 1.14)	0.37	172	0.99 (0.78, 1.27)	0.98	0.97

*Quartiles (Q) of KI67 (Q1, <25 percentile (0–1.49%); Q2, 25–50th percentile (1.50–4.29%); Q3, >50–75th percentile (4.30–10.40%); Q4, >75th percentile (>10.40%)) were derived from the distribution of KI67 scores. ORs and corresponding tests are based on data from all cases i.e. both population and non‐population‐based. All models were adjusted for age and study and no evidence was observed of between‐study heterogeneity in study‐specific OR estimates for nulliparity (*p*‐value = 0.85).

Statistically significant *p*‐values are indicated in bold.

### Case–control comparisons for the associations between nulliparity, BMI, HT use and HR+ tumors defined by levels of KI67 and histologic grade

**Figure 1:**
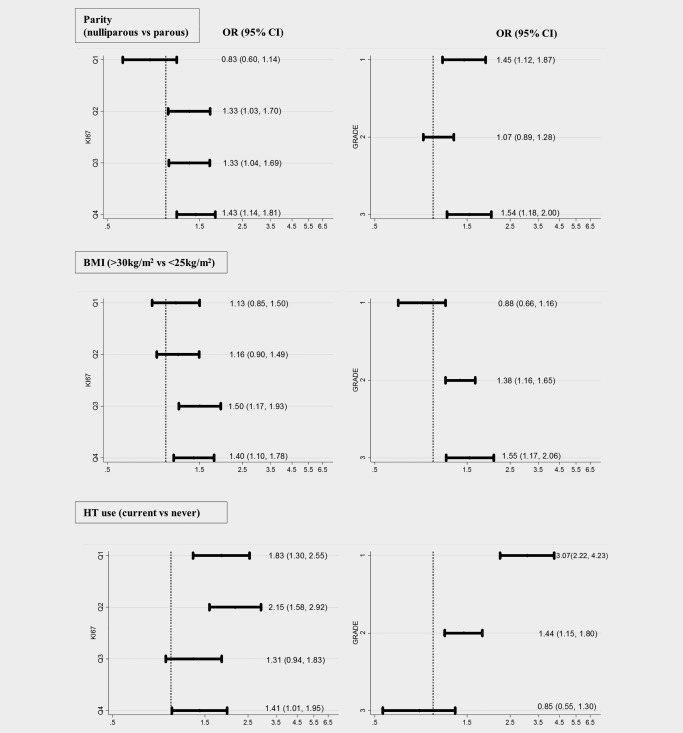
Case–control odds ratios (OR) and 95% confidence intervals (CI) for the associations between parity, BMI, use of combined HT and risk of HR+ tumors defined by levels of histologic grade and tumor proliferation, indicated by KI67. Levels of KI67 defined by quartiles of expression (Q1, <25th percentile (0–1.49%); Q2, 25–50th percentile (1.50–4.29%); Q3, 50–75th percentile (4.30–10.40%); Q4, >75th percentile (>10.40%)). Histologic grade defined as: 1 = well‐differentiated; 2 = moderately differentiated and 3 = poorly differentiated. All models were adjusted for age and study. No evidence was observed of between‐study heterogeneity in study‐specific OR estimates (*p*‐value > 0.05). For more details see Supporting Information Tables S5, S6 and S8.

Case–control comparisons in population‐based studies showed an elevated risk of HR+ tumors with high levels of tumor proliferation among nulliparous women (Fig. 1 and in Supporting Information Table S5; *p*‐value for between‐study heterogeneity = 0.78). Furthermore, as shown in Figure 1 and in Supporting Information Table S6, obesity amongst women older than 50 years of age was associated with elevated risks of high but not low grade tumors (*p*‐value for between‐study heterogeneity = 0.76). Among women younger than 50 years of age (Supporting Information Table S7), we observed obesity to be associated with reduced risk of breast cancer across all levels of histologic grade, this association was however weaker for grades 2 and 3 than grade 1 tumors (*p*‐value for between‐study heterogeneity = 0.72). Current use of combined HT was associated with an elevated risk of low but not high grade tumors (Fig. 1 and in Supporting Information Table S8; *p*‐value for between‐study heterogeneity = 0.15). In multivariate analyses with mutual adjustment for the five risk factors that were evaluated in addition to age and study group, nulliparity remained significantly associated with high but not low KI67 expressing tumors [OR (95% CI) = 1.33 (1.02, 1.74); *p*‐value = 0.03 for KI67 Q4 and 0.85 (0.57, 1.25); *p*‐value = 0.40 for KI67 Q1]. Obesity among women ≥50 years of age remained significantly associated with high but not low grade tumors [OR (95% CI) = 1.50 (1.04, 2.18); *p*‐value = 0.03 for grade 3 and 0.82 (0.58, 1.15); *p*‐value = 0.26 for grade 1]. Current use of combined HT remained significantly associated with low but not high grade tumors [3.04 (2.19, 4.21); *p*‐value <0.001 for grade 1 and 0.89 (0.58, 1.38); *p*‐value = 0.61 for grade 3].

When we examined the associations between nulliparity, obesity, HT use and subtypes of HR+ tumors defined by cross‐classification of levels of KI67 and histologic grade (Table 4), we observed nulliparity to be more strongly associated with tumors expressing higher levels of KI67 and this association remained significant after accounting for grade (*p*‐value = 0.04) and was not modified by grade (*p*__interaction_ = 0.37). Grade was determined to be the primary tumor characteristic associated with obesity (*p*‐value = 0.03) and this was regardless of KI67 levels (*p*__interaction_ = 0.59). Furthermore, HT use was more strongly associated with subtypes characterized by being low grade. We observed grade, not KI67, to be the primary tumor characteristic associated with HT use (*p*‐value = 0.008) and there was no evidence to suggest that this association is dependent on levels of KI67 in the tumor (*p*__interaction_ = 0.48).

**Table 4:** Odds ratios (OR) and 95% CI for the associations between parity, obesity, HT and subtypes of HR+ tumors defined by cross‐classification of levels (Q1–Q4) of KI67 and histologic grade

				Parity		Obesity		Combined HT	
				Nulliparous *vs*. parous		Obese *vs*. normal		Current *vs*. never	
Subtype	*N*	KI67	Grade	OR (95% CI)	*p*‐Value	OR (95% CI)	*p*‐Value	OR (95% CI)	*p*‐Value
Controls	11,475			1.00 (referent)		1.00 (referent)		1.00 (referent)	
1	102	Q1	Low	0.74 (0.38, 1.44)	0.38	0.98 (0.53, 1.80)	0.94	**3.88 (2.14, 7.04)**	**<0.0001**
2	155	Q2	Low	**1.56 (1.03, 2.42)**	**0.03**	0.75 (0.46, 1.21)	0.24	**2.91 (1.72, 4.92)**	**<0.0001**
3	123	Q3	Low	**1.68 (1.05, 2.69)**	**0.03**	0.88 (0.52, 1.49)	0.63	**2.08 (1.14, 3.80)**	**0.02**
4	79	Q4	Low	**2.16 (1.24, 3.74)**	**0.006**	0.93 (0.49, 1.77)	0.83	**2.77 (1.41, 5.44)**	**0.003**
5	300	Q1	High	0.84 (0.57, 1.14)	0.35	1.14 (0.83, 1.58)	0.41	1.13 (0.74, 1.73)	0.56
6	370	Q2	High	1.19 (0.88, 1.62)	0.25	1.30 (0.97, 1.76)	0.08	**1.69 (1.19, 2.42)**	**0.004**
7	451	Q3	High	1.28 (0.97, 1.69)	0.08	**1.80 (1.35, 2.39)**	**<0.0001**	1.20 (0.84, 1.72)	0.29
8	553	Q4	High	**1.37 (1.07, 1.76)**	**0.01**	**1.48 (1.14, 1.93)**	**0.003**	1.26 (0.91, 1.77)	0.16
KI67^a^				**1.19 (1.01, 1.39)**	**0.04**	1.09 (0.93, 1.27)	0.22	0.95 (0.79, 1.15)	0.51
Grade^b^				0.75 (0.51, 1.11)	0.12	**1.63 (1.08, 2.46)**	**0.03**	**0.47 (0.30, 0.74)**	**0.008**

Subtypes were defined by cross‐classification of levels (Q1–Q4) of KI67 and histologic grade (low = grade 1 and high = grades 2 and 3).

*p*_interaction = 0.37 for parity, 0.59 for obesity and 0.49 for HT.

a Association between KI67 (high *vs*. low) and parity, obesity and HT after accounting for histologic grade.

b Association between grade (high *vs*. low) and parity, obesity and HT after accounting for KI67.

Statistically significant *p*‐values are indicated in bold.

## Discussion

Findings from analyses including almost 6,000 cases with HR+ tumors provide evidence for heterogeneity within these tumors by histologic grade and level of proliferation. Nulliparity was primarily associated with risk of HR+ tumors with high levels of proliferation defined by KI67; whilst BMI and HT were associated with risk of high and low grade HR+ tumors, respectively.

Epidemiological studies have shown that nulliparity is more consistently associated with increased risk for HR+ than HR− breast cancer.^7^, ^9^, ^29^, ^30^ Our analyses indicate that nulliparity is primarily associated with an elevated risk of HR+ tumors with high levels of proliferation, which is consistent with findings from a previous prospective study.^31^ These findings could reflect parity‐related mechanisms influencing the proliferative potential of mammary epithelial cells via the induction of terminal differentiation.^32^ This is in keeping with animal studies that show pregnancy‐mediated persistent increase in the differentiated state of the mammary gland, in addition to reduction in epithelial cell proliferation mediated, at least in part, by the downregulation of growth factors and the upregulation of growth‐inhibitory molecules.^33^

Postmenopausal obesity is associated with an elevated risk of breast cancer that is more consistent for the HR+ subtype.^34^ Consistent with our findings, previous studies have reported a higher frequency of high grade^21^, ^22^, ^23^ and large^35^ tumors amongst obese women; however, it is unclear whether these reported observations are driven by grade, tumor size or proliferation since these features are correlated but seldom studied simultaneously. Our analyses indicate that grade is the primary tumor characteristics related to obesity. Several biological pathways involving estrogen metabolism,^36^, ^37^ insulin resistance, inflammation and altered adipokine and cytokine production, have been proposed to mediate the obesity‐cancer link.^38^ It is plausible that obesity‐induced systemic and/or intra‐tumoral inflammation may contribute to the emergence, via cancer immunoediting^39^ and/or noncellular mechanisms,^40^ of aggressive forms of breast tumors. Further studies will be required to unravel the mechanisms underpinning the relationship between BMI and breast cancer histopathological characteristics.

Use of combined HT has been shown in epidemiological studies to be consistently associated with tumors with favorable biological profile including HR+, lobular or tubular morphology, small and low grade tumors.^35^, ^41^, ^42^, ^43^, ^44^ In line with these reports, we found an association between HT and HR+ low grade tumors, that is independent of KI67. The current analysis includes data from a previously published study (PBCS) where we reported an association with low grade but did not measure KI67.^35^ HT use is known to be more strongly associated with the invasive lobular cancers, typically low grade and low proliferating,^45^, ^46^ than with no‐special‐type (NST) invasive ductal carcinomas, which represent 50–70% of all invasive cancers. However, our analyses indicated that HT use predisposes similarly to low grade tumors, independently of morphology. More active screening among HT users may lead to detection of tumors with more favorable features including being low grade. Due to lack of information on screening history and mode of detection, we were unable to directly examine the impact of screening on our findings. We did this indirectly, by using tumor size as proxy for mode of detection and observed HT to be associated with low grade tumors regardless of tumor size (*p*‐value for heterogeneity = 0.78). Thus, our findings could reflect a biological role for HT in influencing tumor behavior; however, further studies directly accounting for screening history and mode of detection will be needed to clarify relationships. Postmenopausal obesity has been shown to increase the risk of breast cancer only among women who do not take HT.^47^, ^48^ We stratified our case–case analyses by HT use and our results remained essentially the same even though numbers of cases were small.

An important strength of this analysis is that we centrally generated continuous measures of tumor proliferation using automated digital‐pathology algorithms to score KI67. As we previously showed, this provides standardized, highly reproducible measures of KI67 with good agreements with pathologists' quantitative and semi‐quantitative scores.^26^ This allowed us to evaluate dose–response relationships using quartiles, rather than arbitrary dichotomous categories of tumor proliferation. In addition, data on other pathology markers enabled us to evaluate breast cancer risk factors in relation to both KI67 and grade in the context of tumor size and morphology.

KI67 scores were obtained from TMAs that are generally lower than those obtained on whole sections.^49^ In addition, we used an automated system to generate KI67 scores that are usually lower than visual scores, regardless of whether measurement was made on TMAs or whole sections.^26^, ^50^ Thus, our scores for proliferation were lower than what is typically obtained for whole sections or following visual scoring on TMAs. Nonetheless, measurements from different sources are generally well correlated and unlikely to substantially affect the ranking of cases in relation to levels of KI67 used in our analyses. Measurement error is a notable limitation for KI67 but automated methods are highly reproducible and show adequate accuracy in relation to standardized pathologists' scores.^26^, ^51^ Furthermore, measurement error is unlikely to be differential with respect to risk factors, and therefore it would tend to under‐rather than over‐estimate odds ratios. Histologic grade tends to have low reproducibility within and between pathologists,^52^ however, this error is also likely to be non‐differential with respect to risk factors. Moreover, the consistency of our results with those of others who have assessed breast cancer risk factors in relation to KI67 and grade together,^21^, ^22^, ^23^, ^31^ suggest that measurement error is unlikely to explain our findings.

Our analyses comprised multiple studies with different study designs, including population and non‐population‐based studies: non‐population‐based studies are particularly prone to biases in case–control measures of association since the distribution of exposures amongst controls often does not reflect that in the source population for the cases. To address this, we limited case–control comparisons to population‐based studies only. Tests of heterogeneity of associations by study revealed no evidence of heterogeneity of effect estimates for both case–control and case–case comparisons. Missing data on risk factors were another limitation in our study, particularly for case–control comparisons. To address this, we limited the analysis for each risk factor to studies with data on that risk factor in both cases and controls and used the conventional approach of creating indicators for missing values on each risk factor in our regression models. As sensitivity analyses, we performed multivariate analyses with mutual adjustment for all five risk factors in three studies with complete information on all covariates and our results remained essentially the same.

In conclusion, our findings indicate that the associations between parity, BMI, use of combined HT and risk of HR+ tumors are heterogeneous depending on the levels of histologic grade and proliferation, indicated by KI67. Although correlated, histologic grade and KI67 appear to be distinctly related to breast cancer risk factors. These results provide insights into heterogeneity of HR+ tumors that may be reflective of differences in etiological pathways; however, other factors not evaluated in our study, such as screening, could play a role. Given that grade and proliferation are important prognostic factors in HR+ breast cancer, these findings could have implications for risk prediction of aggressive forms of HR+ tumors. Further studies accounting for multiple correlated tumor characteristics and screening are needed to enable better understanding of these relationships.

### Ethical approval and consent to participate

Each of the individual studies was approved by the local Ethics Committees and written informed consent to participate in the study was obtained from each participant across all study groups. The ESTHER study was approved by the Ethics Committees of the Medical Faculty of the University of Heidelberg and the Medical Association of Saarland. The joint Ethics Committee of Kuopio University and Kuopio University Hospital approved the Kuopio Breast Cancer Project (KBCP). Approval for the MARIE study was obtained from the Ethics Committee of the University of Heidelberg, the Hamburg Medical Council and the Medical Board of the State of Rheinland‐Pfalz. MCBCS study was approved by the Ethics Committee of the Mayo Clinic College of Medicine. The Medical Ethical Review Boards of the Rotterdam Cancer Centre and academic cancer center in Leiden approved the study protocol for ORIGO study. The PBCS study protocol was reviewed and approved by local and the US National Cancer Institute (NCI) IRBs. The RBCS study was approved by the Ethical Committees of the University Hospital Rotterdam, Erasmus University Rotterdam and Leiden University Medical Centre, Leiden. The SEARCH study was approved by the Eastern multi‐center research ethics committee. The kConFab study obtained human ethics approval at all the participating institutions through which subjects are recruited.

## Authors' Contributions

MA and MG‐C conceived and carried out the analysis; MG‐C supervised the work; FD, MA carried out centralized laboratory work and automated scoring on KI67, respectively; PC performed KI67 data management; MA and MG‐C analyzed the data with support from NC; MA, JCC, HRA, NC, MES, MKS, PDP and MGC were members of the initial writing group for the manuscript; MA, FB, HRA, PC, JB, RM, HB, CS, AM, JCC, AR, PS, FJC, PD, RAEMT, CS, JF, MES, JL, SH, MJH, AH, RF, LBK, kConFab, MKB, QW, MJ, MJS, RK, DFE, AJS, MKS, PDP, MG‐C contributed to TMA/data collection, data generation and/or data management. All authors contributed to manuscript development and writing and gave final approval for its submission.

## Supporting information

Supporting Information Figure 1

Supporting Information Tables
