# Supplementary figures and images for: Etiology of hormone receptor positive breast cancer differs by levels of histologic grade and proliferation
Source: Int J Cancer. 2018 Mar 25;143(4):746–57. doi: 10.1002/ijc.31352 (PMC6041155; doi:10.1002/ijc.31352)

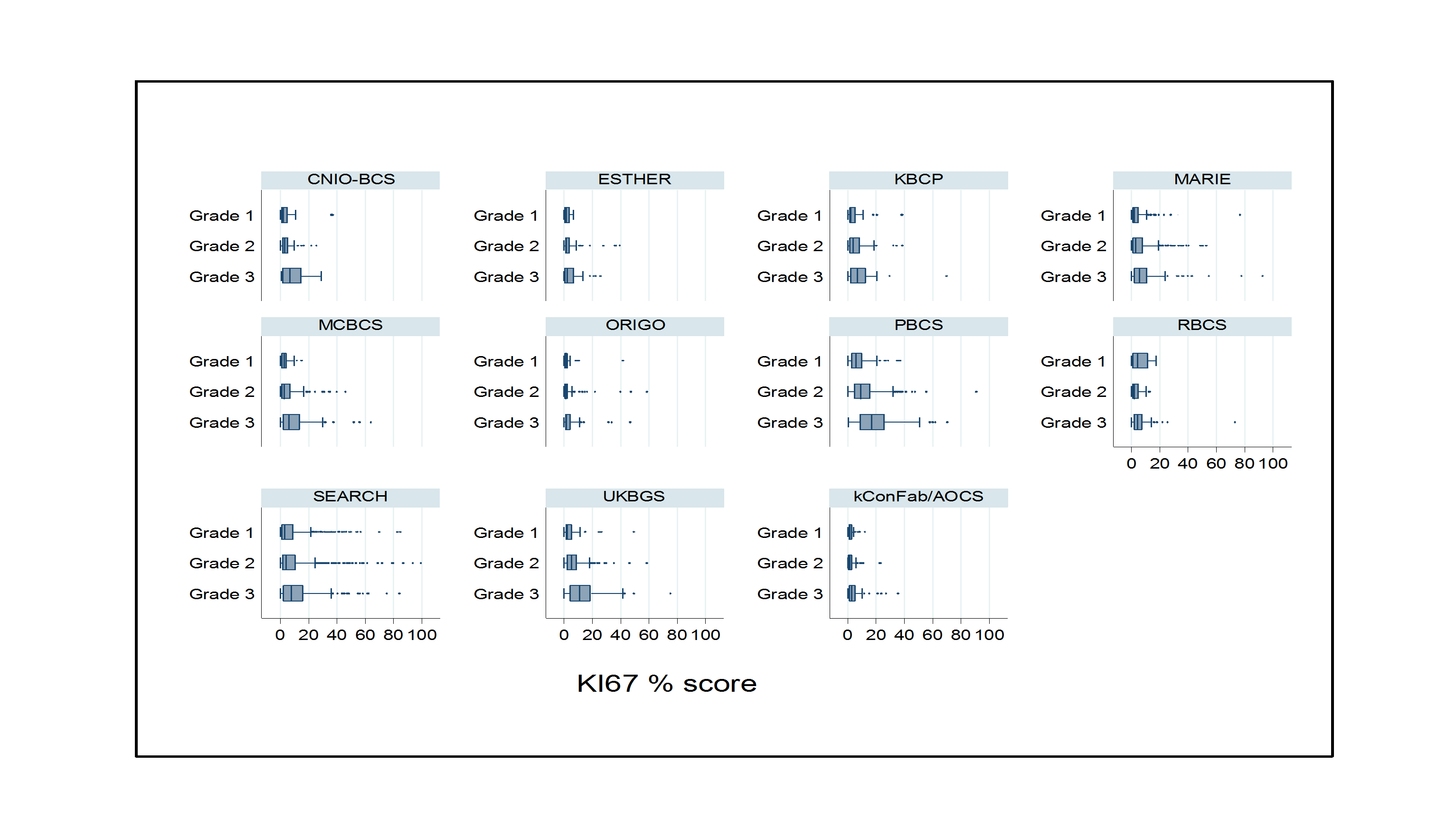

Supplement: Supplementary file 1 — Supporting Information Figure 1 [file IJC-143-746-s001.tif]
