# Supplementary material for: Etiology of hormone receptor positive breast cancer differs by levels of histologic grade and proliferation
Source: Int J Cancer. 2018 Mar 25;143(4):746–57. doi: 10.1002/ijc.31352 (PMC6041155; doi:10.1002/ijc.31352)
Supplement: Supplementary file 2 — Supporting Information Tables [file IJC-143-746-s002.docx]

**Supplementary Table 1**: Description of participating BCAC studies

| Study name | Acronym | Country | Design |
| --- | --- | --- | --- |
|  |  |  |  |
|  |  |  |  |
| Spanish National Cancer Centre Breast Cancer Study | CNIO | Spain | This is a hospital-based case-control study involving populations from Spain. Women with a diagnosis of breast cancer (cases) were recruited between 2000 and 2004 from three Spanish public hospitals including: Monte Naranco hospital in Oviedo, Spain; Fundaciόn Jimenez Diaz and Hospital La Paz. The latter two hospitals are in Madrid, Spain. A few cases were recruited from the Spanish National Cancer Centre family cancer clinic for genetic testing. Women free of breast cancer were recruited as controls from the Menopause Research Centre at the Instituto Palacios in Madrid, Spain and from the Spanish National Centre family cancer clinic for genetic testing (1). |
|  |  |  |  |
| ESTHER Breast cancer study | ESTHER | Germany | This is a population-based case-control study. Cases were women between the ages of 50-75 years with histologically diagnosed breast cancer in all hospitals in the state of Saarland, Germany from 2001 to 2003 that were recruited during their first stay in the hospital. Controls were cancer free women from the general population (2). |
|  |  |  |  |
| Kuopio Breast Cancer Project | KBCP | Finland | This is a population-based case-control study on diet and breast cancer. Cases were recruited from among women who were invited to the Kuopio University Hospital following an initial diagnosis of breast lump or suspected breast disease between April 1990 and December 1995. Of these women, all those with histologically confirmed breast cancer were recruited into the study. During the enrolment period, about 350 women with a diagnosis of breast lump or suspected breast disease were recruited into the Kuopio study annually, out of these, an annual average of 85 breast cancers were diagnosed. Controls were randomly selected from the population register covering the same geographical region and individually matched (±5 years) with cases (3). |
|  |  |  |  |
|  |  |  |  |
| Kathleen Cuningham Foundation Consortium for Familial Breast Cancer | kConFab | Australia and New Zealand | This is a mixed hospital and population-based case-control study that is aimed at collecting relevant data and biological samples from families with cases of breast and/or ovarian cancer. Starting 1997, several individuals including those affected (cases) and unaffected (controls) by breast cancer have been recruited into the study from family cancer clinics around Australia and New Zealand. All reports of cancer in a family are verified through a variety of means including the medical records, state-based cancer registries and by other systematic searches of Australian cancer registry records. Familial breast cancer patients were recruited in the clinics while controls were women from a population-based case-control study of ovarian cancer (4). |
|  |  |  |  |
| Mammary Carcinoma Risk Factor Investigation. |  |  |  |
|  | MARIE | Germany | This is a population-based case-control study of breast cancer in Northern and Southern Germany. Cases from this study were incident and prevalent cases diagnosed from 2001‐2005 in the study region of Hamburg in Northern Germany and from 2002‐2005 in the study region of Rhein‐Neckar‐Karlsruhe in Southern Germany. Controls were recruited from 2002 to 2006 and were randomly drawn from population registries and frequency matched by birth year and study region to the cases (5). |
|  |  |  |  |
|  |  |  |  |
| Mayo Clinic Breast Cancer Study | MCBCS | USA | This is a hospital-based case-control study in the United States of America. Cases are unselected, clinic based, series of breast cancer patients diagnosed within the previous 6 months with no prior history of cancer (except non-melanoma skin cancer) who were seen in the division of Medical Oncology between February 1, 2001 and June 2005. Controls were women seen at the Mayo Clinic for a non-specific medical exam in the Internal Medicine department and were frequency matched to cases on region of residence (Iowa, Minnesota, Wisconsin, North or South Dakota, Illinois) and age (5-year age group) (6). |
|  |  |  |  |
| Leiden University Medical Centre Breast Cancer Study | ORIGO | Netherlands | This is a hospital-based case-control study in the Netherlands. Cases were a consecutive series of breast cancer cases unselected for family history recruited from three centres in south west Netherlands (including two academic cancer centres in Leiden and Rotterdam, and one general hospital in Leiden) between October 1, 1996 and July 1, 2002. Controls were other hospital patients who were in the hospital for other causes unrelated to breast cancer (7). |
|  |  |  |  |
| NCI Polish Breast Cancer Study | PBCS | Poland | This is a population-based case-control study set in Poland over a three-year period between 2000 and 2003. Cases were women with histologically or cytologically confirmed breast cancer and between the ages of 20 and 74 years. Controls were randomly selected using the Polish electronic system – a directory of all Polish residents – and frequency matched to cases on city and age in 5-year categories (8). |
|  |  |  |  |
| Rotterdam Breast Cancer Study | RBCS | Netherlands | This is a hospital based case-control study based in the Netherlands. Cases were ascertained through the familial cancer clinic of the Erasmus MC between 1993 and 2005 and include familial non-BRCA1/2 cases that were diagnosed with histologically confirmed breast cancer between 1983 to 2008. Controls were other hospital patients who received clinical genetic counseling for cystic fibrosis and were all non-carriers of a CTFR mutation (9). |
|  |  |  |  |
| Study of Epidemiology and Risk Factors in Cancer Heredity | SEARCH | UK | This is a mixed hospital and population-based study in the UK. Cases were ascertained through the East Anglian Cancer Registry (including the counties of Cambridgeshire, Norfolk, Suffolk, Bedfordshire, Hertfordshire and Essex) and included women diagnosed with invasive breast cancer below the age of 55 years between 1991 and mid-1996 and still alive in 1996 when the study began together with women with invasive breast cancer diagnosed at age <70 years from the mid 1996 onwards. Controls were women recruited through general practices (GPs) that recruited the cases (10). |
|  |  |  |  |
| UK Breakthrough Generations Study | BGS | UK | This is a prospective cohort study in the United Kingdom. It began in 2003 and has so far recruited over 110,000 women. The age range of subjects at recruitment is 16-102 and subjects who are enrolled in the study volunteer to enroll a new generation of study subjects: these can be friends and/or family members. Extensive questionnaire information was obtained from these patients including blood samples and anthropometric measurements and this process is repeated every three and half years. Cases in the BCAC study are those individuals who developed breast cancer during the follow-up period whilst controls are age-matched women in the cohort who had not developed breast cancer (11). |

**Supplementary Table 2**: Availability of risk factor data by study group for cases and controls

|  |  |  | **Menarche** | |  | **Parity** | |  | **BMI** | |  | **HT** | |  | **Family history** | |
| --- | --- | --- | --- | --- | --- | --- | --- | --- | --- | --- | --- | --- | --- | --- | --- | --- |
| **Study** | **Total cases**  **(HR+)** | **Total controls** | **Cases** | **Controls** |  | **Cases** | **Controls** |  | **Cases** | **Controls** |  | **Cases** | **Controls** |  | **Cases** | **Controls** |
| CNIO | 83 | 1,012 | 74 | 631 |  | 47 | 622 |  | 0 | 638 |  | 0 | 0 |  | 0 | 0 |
| ESTHER | 175 | 507 | 169 | 498 |  | 167 | 473 |  | 167 | 494 |  | 0 | 0 |  | 147 | 418 |
| KBCP | 202 | 492 | 191 | 443 |  | 197 | 467 |  | 192 | 443 |  | 0 | 0 |  | 197 | 492 |
| MARIE | 609 | 5,282 | 518 | 4,468 |  | 605 | 5,282 |  | 604 | 5,266 |  | 600 | 5,239 |  | 590 | 5146 |
| MCBCS | 382 | 2,428 | 359 | 2,122 |  | 373 | 2,284 |  | 361 | 2,255 |  | 262 | 1,428 |  | 355 | 2218 |
| ORIGO | 223 | 1,469 | 147 | 0 |  | 160 | 0 |  | 199 | 0 |  | 0 | 0 |  | 39 | 0 |
| PBCS | 889 | 2,379 | 879 | 2,351 |  | 885 | 2,379 |  | 884 | 2,376 |  | 837 | 2,258 |  | 884 | 2379 |
| RBCS | 131 | 734 | 32 | 0 |  | 121 | 0 |  | 0 | 0 |  | 0 | 0 |  | 125 | 0 |
| SEARCH | 2,757 | 8,155 | 1,870 | 5,346 |  | 1,929 | 5,576 |  | 1,912 | 4,057 |  | 0 | 0 |  | 1,959 | 5785 |
| UKBGS | 301 | 2,815 | 121 | 420 |  | 136 | 462 |  | 132 | 451 |  | 0 | 0 |  | 136 | 462 |
| kConFab | 153 | 1,008 | 137 | 517 |  | 142 | 273 |  | 135 | 184 |  | 0 | 0 |  | 118 | 779 |
| Total | 5,905 | 26,281 | 4,497 | 16,796 |  | 4,762 | 17,818 |  | 4,611 | 16,164 |  | 1,699 | 8,925 |  | 4,575 | 17,679 |

Green shaded areas indicate situations in which a study had data only on cases or controls but not on both; yellow shaded areas indicate situations in which data on both cases and controls were not available; grey shaded areas indicate situations in which data on a specific risk factor were available for both cases and controls. The difference in the total numbers of cases and controls per study and those in the shaded areas represent missing values on each risk factor. BMI = Body Mass Index, HT = Hormone replacement therapy.

**Supplementary Table 3**: Availability of data on KI67 and histologic grade for HR+ tumors by study group

|  |  |  | **All cases** |  | **Grade** |  | **KI67** |
| --- | --- | --- | --- | --- | --- | --- | --- |
| **Study** |  |  | **No.** |  | **No.** |  | **No.** |
| CNIO |  |  | 83 |  | 65 |  | 83 |
| ESTHER |  |  | 175 |  | 167 |  | 175 |
| KBCP |  |  | 202 |  | 196 |  | 202 |
| MARIE |  |  | 609 |  | 599 |  | 609 |
| MCBCS |  |  | 382 |  | 342 |  | 382 |
| ORIGO |  |  | 223 |  | 199 |  | 223 |
| PBCS |  |  | 889 |  | 872 |  | 889 |
| RBCS |  |  | 131 |  | 100 |  | 131 |
| SEARCH |  |  | 2,757 |  | 2,303 |  | 2,757 |
| UKBGS |  |  | 301 |  | 299 |  | 301 |
| kConFab |  |  | 153 |  | 132 |  | 153 |
| Total |  |  | 5,905 |  | 5,274 |  | 5,905 |

All studies had data on KI67 and histologic grade. Differences between the total number of cases and the numbers recorded under each variable per study represent missing values on that variable. Data on hormone receptor status and histological grade were obtained from clinical records whilst those on KI67 were centrally generated using an automated scoring algorithm at the ICR in London.

**Supplementary Table 4**: Case-case odds ratios (OR) and 95% confidence intervals (CI) for the associations between BMI, HT and histologic grade for tumors stratified by size, intake of HT and morphology.

|  |  |  |  | |  |  |  |  |
| --- | --- | --- | --- | --- | --- | --- | --- | --- |
|  |  | **Grade 1 (comparison)** | **Grade 2** | | | **Grade 3** | | |
|  |  | **N** | **N** | **OR (95% CI)** | **p-value** | **N** | **OR (95% CI)** | **p-value** |
|  |  |  |  |  |  |  |  |  |
|  |  |  | **Size < 2cm** | |  |  |  |  |
| **BMI** |  |  |  |  |  |  |  |  |
| ≤ 25 kg/m^2^ |  | 329 | 443 | 1.00 (referent) |  | 151 | 1.00 (referent) |  |
| 25-30 kg/m^2^ |  | 266 | 440 | 1.32 (1.06, 1.65) | 0.01 | 93 | 0.98 (0.71, 1.37) | 0.93 |
| >30 kg/m^2^ |  | 129 | 259 | 1.63 (1.25, 2.12) | <0.01 | 55 | 1.30 (0.88, 1.93) | 0.19 |
|  |  |  |  |  |  |  |  |  |
| **HT** |  |  |  |  |  |  |  |  |
| Never |  | 137 | 279 | 1.00 (referent) |  | 41 | 1.00 (referent) |  |
| Current |  | 44 | 46 | 0.49 (0.29, 0.83) | 0.008 | 7 | 0.31 (0.09, 1.11) | 0.07 |
|  |  |  |  |  |  |  |  |  |
|  |  |  | **Size ≥ 2cm** | |  |  |  |  |
| **BMI** |  |  |  |  |  |  |  |  |
| ≤ 25 kg/m^2^ |  | 72 | 220 | 1.00 (referent) |  | 116 | 1.00 (referent) |  |
| 25-30 kg/m^2^ |  | 65 | 285 | 1.44 (0.97, 2.15) | 0.07 | 144 | 1.33 (0.86, 2.07) | 0.19 |
| >30 kg/m^2^ |  | 47 | 225 | 1.65 (1.06, 2.55) | 0.02 | 103 | 1.49 (0.92, 2.41) | 0.11 |
|  |  |  |  |  |  |  |  |  |
| **HT** |  |  |  |  |  |  |  |  |
| Never |  | 47 | 250 | 1.00 (referent) |  | 85 | 1.00 (referent) |  |
| Current |  | 9 | 17 | 0.35 (0.12, 1.00) | 0.05 | 5 | 0.35 (0.10, 1.25) | 0.11 |
|  |  |  |  |  |  |  |  |  |
|  |  |  | **Never users of HT** | |  |  |  |  |
| **BMI** |  |  |  |  |  |  |  |  |
| ≤ 25 kg/m^2^ |  | 80 | 211 | 1.00 (referent) |  | 60 | 1.00 (referent) |  |
| 25-30 kg/m^2^ |  | 77 | 269 | 1.36 (0.94, 1.95) | 0.09 | 76 | 1.39 (0.87, 2.21) | 0.16 |
| >30 kg/m^2^ |  | 68 | 239 | 1.43 (0.98, 2.10) | 0.06 | 71 | 1.64 (1.01, 2.66) | 0.04 |
|  |  |  |  |  |  |  |  |  |
|  |  |  | **Current users of HT** | |  |  |  |  |
| **BMI** |  |  |  |  |  |  |  |  |
| ≤ 25 kg/m^2^ |  | 39 | 60 | 1.00 (referent) |  | 14 | 1.00 (referent) |  |
| 25-30 kg/m^2^ |  | 40 | 60 | 1.08 (0.60, 1.92) | 0.799 | 14 | 1.11 (0.46, 2.67) | 0.814 |
| >30 kg/m^2^ |  | 10 | 29 | 2.48 (1.07, 5.75) | 0.035 | 5 | 2.00 (0.56, 7.05) | 0.280 |
|  |  |  |  |  |  |  |  |  |
|  |  |  | **Invasive ductal carcinoma** | |  |  |  |  |
| **HT** |  |  |  |  |  |  |  |  |
| Never |  | 25 | 153 | 1.00 (referent) |  | 51 | 1.00 (referent) |  |
| Current |  | 11 | 14 | 0.23 (0.09, 0.58) | 0.002 | 4 | 0.16 (0.04, 0.57) | 0.005 |

**Supplementary Table 5**: Case-control odds ratios (OR) and 95% confidence intervals (CI) for the associations between parity and risk of HR+ tumors defined by levels of KI67 and histologic grade

|  |  |  |  |  | **Case-control** | | |
| --- | --- | --- | --- | --- | --- | --- | --- |
|  |  | **Nulliparous (N)** |  | **Parous (N)** |  | **Nulliparous vs parous** | |
| **Characteristic** |  | **case/control** |  | **case/control** |  | **OR (95% CI)** | **p-value** |
|  |  |  |  |  |  |  |  |
| **Overall controls** |  | 1,221 |  | 7,842 |  |  |  |
|  |  |  |  |  |  |  |  |
| **KI67*** |  |  |  |  |  |  |  |
| Q1 |  | 44 |  | 349 |  | 0.83 (0.60, 1.14) | 0.52 |
| Q2 |  | 82 |  | 408 |  | 1.33 (1.03, 1.70) | 0.02 |
| Q3 |  | 85 |  | 441 |  | 1.33 (1.04, 1.69) | 0.02 |
| Q4 |  | 99 |  | 482 |  | 1.43 (1.14, 1.81) | 0.002 |
|  |  |  |  |  |  |  |  |
| **GRADE** |  |  |  |  |  |  |  |
| Grade 1 |  | 76 |  | 351 |  | 1.45 (1.12, 1.87) | 0.005 |
| Grade 2 |  | 156 |  | 979 |  | 1.07 (0.89, 1.28) | 0.48 |
| Grade 3 |  | 73 |  | 314 |  | 1.54 (1.18, 2.00) | 0.002 |
|  |  |  |  |  |  |  |  |

Analysis was restricted to population-based studies only to minimize bias.

In the case-case comparison, we observed differences in the association between nulliparity and HR+ tumors defined by levels of KI67 but not histologic grade

*p-value for between-study heterogeneity in OR estimates for nulliparity in relation to KI67 = 0.78.

**Supplementary Table 6**: Case-control odds ratios (OR) and 95% confidence intervals (CI) for the associations between BMI in women ≥50 years and risk of HR+ tumors defined by levels of KI67 and histologic grade

|  |  |  | **Case-control** | |  | **Case-control** | |
| --- | --- | --- | --- | --- | --- | --- | --- |
|  | **<25kg/m^2^ (N)** | **25-30kg/m^2^ (N)** | **25-30 vs <25kg/m^2^** | | **>30kg/m^2^ (N)** | **>30 vs <25kg/m^2^** | |
| **Characteristic** | **Case/control** | **Case/control** | **OR (95% CI)** | **p-value** | **Case/control** | **OR (95% CI)** | **p-value** |
|  |  |  |  |  |  |  |  |
| **Overall controls** | 2,775 | 3,028 |  |  | 2,005 |  |  |
|  |  |  |  |  |  |  |  |
| **KI67** |  |  |  |  |  |  |  |
| Q1 | 115 | 146 | 1.28 (1.00, 1.64) | 0.04 | 82 | 1.13 (0.85, 1.50) | 0.41 |
| Q2 | 138 | 156 | 1.13 (0.90, 1.43) | 0.29 | 115 | 1.16 (0.90, 1.49) | 0.24 |
| Q3 | 109 | 178 | 1.53 (1.21, 1.94) | 3.3×10^-4^ | 140 | 1.50 (1.17, 1.93) | 0.001 |
| Q4 | 110 | 161 | 1.29 (1.02, 1.63) | 0.03 | 149 | 1.40 (1.10, 1.78) | 0.007 |
|  |  |  |  |  |  |  |  |
| **GRADE*** |  |  |  |  |  |  |  |
| Grade 1 | 109 | 142 | 1.16 (0.91, 1.48) | 0.24 | 86 | 0.88 (0.66, 1.16) | 0.38 |
| Grade 2 | 268 | 363 | 1.31 (1.11, 1.54) | 0.001 | 29 | 1.38 (1.16, 1.65) | 2.6×10^-4^ |
| Grade 3 | 89 | 121 | 1.34 (1.02, 1.75) | 0.03 | 98 | 1.55 (1.17, 2.06) | 0.003 |
|  |  |  |  |  |  |  |  |

Analysis was restricted to population-based studies only to minimize bias.

In the case-case comparison, we observed differences in the association between BMI and HR+ tumors defined by levels of histologic grade but not KI67

*p-value for between-study heterogeneity in OR estimates for obesity in relation to histologic grade = 0.76

**Supplementary Table 7**: Case-control odds ratios and 95% confidence intervals (CI) for the associations between BMI in women < 50 years of age and risk of HR+ tumors defined by levels of KI67 and histologic grade

|  |  |  | **Case-control** | |  | **Case-control** | |
| --- | --- | --- | --- | --- | --- | --- | --- |
|  | **<25kg/m^2^ (N)** | **25-30kg/m^2^ (N)** | **25-30 vs <25kg/m^2^** | | **>30kg/m^2^ (N)** | **>30 vs <25kg/m^2^** | |
| **Characteristic** | **Case/control** | **Case/control** | **OR (95% CI)** | **p-value** | **Case/control** | **OR (95% CI)** | **p-value** |
|  |  |  |  |  |  |  |  |
| **Overall controls** | 542 | 431 |  |  | 249 |  |  |
|  |  |  |  |  |  |  |  |
| **KI67** |  |  |  |  |  |  |  |
| Q1 | 23 | 21 | 0.80 (0.48, 1.32) | 0.37 | 2 | 0.13 (0.03, 0.54) | 0.005 |
| Q2 | 38 | 29 | 0.58 (0.38, 0.90) | 0.01 | 10 | 0.36 (0.19, 0.71) | 0.003 |
| Q3 | 54 | 29 | 0.49 (0.32, 0.75) | 0.001 | 15 | 0.46 (0.26, 0.80) | 0.006 |
| Q4 | 79 | 65 | 0.81 (0.59, 1.12) | 0.19 | 15 | 0.33 (0.19, 0.58) | 1.1×10^-4^ |
|  |  |  |  |  |  |  |  |
| **GRADE*** |  |  |  |  |  |  |  |
| Grade 1 | 52 | 27 | 0.46 (0.30, 0.72) | 0.001 | 8 | 0.24 (0.12, 0.50) | 6.2×10^-4^ |
| Grade 2 | 98 | 85 | 0.72 (0.55, 0.96) | 0.02 | 24 | 0.36 (0.23, 0.57) | 9.5×10^-6^ |
| Grade 3 | 39 | 27 | 0.69 (0.44, 1.09) | 0.11 | 10 | 0.47 (0.24, 0.92) | 0.02 |
|  |  |  |  |  |  |  |  |

Analysis was restricted to population-based studies only to minimize bias.

In the case-case comparison, we observed differences in the association between BMI and HR+ tumors defined by levels of histologic grade but not KI67

*p-value for between-study heterogeneity in OR estimates for obesity in relation to histologic grade = 0.72.

**Supplementary Table 8**: Case-control odds ratios (OR) and 95% confidence intervals (CI) for the associations between use of combined HT formulations and risk of HR+ tumors defined by levels of KI67 and histologic grade

|  |  |  | **Case-control** | |  | **Case-control** | |
| --- | --- | --- | --- | --- | --- | --- | --- |
|  | **Never (N)** | **Former (N)** | **Former vs Never** | | **Current (N)** | **Current vs Never** | |
| **Characteristic** | **Case/control** | **Case/control** | **OR (95% CI)** | **p-value** | **Case/control** | **OR (95% CI)** | **p-value** |
|  |  |  |  |  |  |  |  |
| **Overall controls** | 4,133 | 821 |  |  | 630 |  |  |
|  |  |  |  |  |  |  |  |
| **KI67** |  |  |  |  |  |  |  |
| Q1 | 116 | 25 | 1.12 (0.72, 1.75) | 0.61 | 56 | 1.83 (1.30, 2.55) | 4.4×10^-4^ |
| Q2 | 168 | 27 | 1.09 (0.71, 1.66) | 0.70 | 68 | 2.15 (1.58, 2.92) | 1.1×10^-6^ |
| Q3 | 239 | 28 | 0.91 (0.60, 1.37) | 0.65 | 50 | 1.31 (0.94, 1.83) | 0.105 |
| Q4 | 263 | 23 | 0.77 (0.49, 1.21) | 0.26 | 51 | 1.41 (1.01, 1.95) | 0.04 |
|  |  |  |  |  |  |  |  |
| **GRADE*** |  |  |  |  |  |  |  |
| Grade 1 | 144 | 25 | 1.37 (0.87, 2.14) | 0.16 | 70 | 3.07 (2.22, 4.23) | 8.1×10^-12^ |
| Grade 2 | 488 | 62 | 0.93 (0.70, 1.24) | 0.62 | 122 | 1.44 (1.15, 1.80) | 0.001 |
| Grade 3 | 146 | 15 | 0.60 (0.35, 1.03) | 0.06 | 27 | 0.85 (0.55, 1.30) | 0.46 |
|  |  |  |  |  |  |  |  |

Analysis was restricted to population-based studies only to minimize bias.

In the case-case comparison, we observed differences in the association between BMI and HR+ tumors defined by levels of histologic grade but not KI67

*p-value for between-study heterogeneity in OR estimates for current use of HT in relation to histologic grade = 0.15.

**References**

1. Milne RL, Ribas G, González-Neira A, Fagerholm R, Salas A, González E, et al. ERCC4 associated with breast cancer risk: a two-stage case-control study using high-throughput genotyping. Cancer research. 2006;66(19):9420-7.

2. Widschwendter M1, Lichtenberg-Frate H, Hasenbrink G, Schwarzer S, Dawnay A, Lam A, Menon U, Apostolidou S, Raum E, Stegmaier C, Jacobs IJ, Brenner. Serum oestrogen receptor alpha and beta bioactivity are independently associated with breast cancer: a proof of principle study. Br J Cancer. 2009 Jul 7;101(1):160-5.

3. Hartikainen JM, Tuhkanen H, Kataja V, Dunning AM, Antoniou A, Smith P, et al. An Autosome-Wide Scan for Linkage Disequilibrium–Based Association in Sporadic Breast Cancer Cases in Eastern Finland: Three Candidate Regions Found. Cancer Epidemiology Biomarkers & Prevention. 2005;14(1):75-80.

4. Mann G, Thorne H, Balleine R, Butow P, Clarke C, Edkins E, et al. Analysis of cancer risk and BRCA1 and BRCA2 mutation prevalence in the kConFab familial breast cancer resource. Breast Cancer Research. 2006;8(1):R12.

5. Flesch-Janys D, Slanger T, Mutschelknauss E, Kropp S, Obi N, Vettorazzi E, et al. Risk of different histological types of postmenopausal breast cancer by type and regimen of menopausal hormone therapy. International Journal of Cancer. 2008;123(4):933-41.

6. Olson JE, Ingle JN, Ma CX, Pelleymounter LL, Schaid DJ, Pankratz VS, et al. A comprehensive examination of CYP19 variation and risk of breast cancer using two haplotype-tagging approaches. Breast Cancer Res Treat. 2007;102(2):237-47.

7. De Bock G, Schutte M, Krol-Warmerdam E, Seynaeve C, Blom J, Brekelmans C, et al. Tumour characteristics and prognosis of breast cancer patients carrying the germline CHEK2* 1100delC variant. Journal of medical genetics. 2004;41(10):731-5.

8. García-Closas M, Egan KM, Newcomb PA, Brinton LA, Titus-Ernstoff L, Chanock S, et al. Polymorphisms in DNA double-strand break repair genes and risk of breast cancer: two population-based studies in USA and Poland, and meta-analyses. Human genetics. 2006;119(4):376-88.

9. L.C. Verhoog, A.M.W. van den Ouweland, E. Berns, M.M. van Veghel-Plandsoen, I.L. van Staveren, A. Wagner, C.C.M. Bartels, M.M.A. Tilanus-Linthorst, P. Devilee, C. Seynaeve, D.J.J. Halley, M.F. Niermeijer, J.G.M. Klijn, H. Meijers-Heijboer, Large regional differences in the frequency of distinct mutations in 517 Dutch breast and/or ovarian cancer families, European Journal of Cancer, Volume 37, Issue 16, 2001, Pages 2082-2090, ISSN 0959-8049, http://dx.doi.org/10.1016/S0959-8049(01)00244-1.

10. Lesueur F, Pharoah PD, Laing S, Ahmed S, Jordan C, Smith PL, et al. Allelic association of the human homologue of the mouse modifier Ptprj with breast cancer. Human molecular genetics. 2005;14(16):2349-56.

11. Swerdlow A, Jones M, Schoemaker M, Hemming J, Thomas D, Williamson J, et al. The Breakthrough Generations Study: design of a long-term UK cohort study to investigate breast cancer aetiology. British journal of cancer. 2011;105(7):911-7.
